# Medical Department of the Pennsylvania College

**Published:** 1852-07

**Authors:** 


*Medical Department of Pennsylvania College.*—We learn from the annual announcement of this flourishing Institution, that the vacancy in the chair of Anatomy, occasioned by the decease of Dr. Wm. R. Grant, has been filled by the election of Dr. J. M. Allen, formerly Demonstrator of Anatomy in Jefferson Medical College, and Lecturer on Anatomy in the Philadelphia Association for Medical Instruction. Dr. John J. Reese has been appointed to the chair of Medical Chemistry and Pharmacy, made vacant by the resignation of Professor Atlee. A chair of Institutes of Medicine has recently been created in the school, and filled by the appointment of Dr. Francis G. Smith, the able senior editor of the Medical Examiner, and Lecturer on Physiology in the Philadelphia Association for Medical Instruction.

These appointments must give great satisfaction to the friends of the institution; and thus strengthened, the school can scarcely fail to attract to its halls, a fair proportion of the immense number of medical students who every year seek instruction at Philadelphia.

The lectures will commence the 11th of October, and continue until the 1st of March, making the course upwards of four months and a half.

